# The Chromosomal Organization of *Isleria hauxwelli* and Its Meaning for the Karyotypic Evolution in Thamnophilidae (Passeriformes)

**DOI:** 10.1002/age.70210

**Published:** 2026-10-02

**Authors:** Paulo Victor De Moraes Ferreira, Talita Fernanda Augusto Ribas, Ane Nobre Monteiro, Lucas Guilherme de Silva Tembra, Milla de Andrade Machado, Patricia C. M. O'Brien, Malcolm Andrew Ferguson‐Smith, Fengtang Yang, Cleusa Yoshiko Nagamachi, Julio Cesar Pieczarka

**Affiliations:** ^1^ Laboratório de Citogenética, Centro de Estudos Avançados da Biodiversidade, ICB Universidade Federal do Pará, PCT‐Guamá Belém Pará Brazil; ^2^ Instituto de Biodiversidade e Florestas Universidade Federal do Oeste do Pará Santarém Pará Brazil; ^3^ Laboratório de Genética e Biodiversidade, Instituto de Ciências da Educação Universidade Federal do Oeste do Pará Santarém Pará Brazil; ^4^ Cambridge Resource Centre for Comparative Genomics, Department of Veterinary Medicine University of Cambridge Cambridge UK; ^5^ School of Life Sciences and Medicine, Shandong University of Technology Zibo Shandong People's Republic of China

## Abstract

The order Passeriformes, the most speciose avian order, includes over 6000 species (~60% of all birds). The Amazon Basin alone hosts > 900 bird species, with Passeriformes representing > 500 of them. The family Thamnophilidae comprises ~200 insectivorous species, accounting for 15% of Neotropical passerine richness. Within Thamnophilinae, *Isleria* is the least speciose genus, containing only two species. It was erected after phylogenetic analyses revealed *Myrmotherula* as non‐monophyletic. To advance knowledge of chromosomal evolution in Passeriformes, we analyzed the karyotype of *Isleria hauxwelli* using molecular cytogenetics. We found a diploid number of 2n = 84 and a fundamental number (FN) of 92. The BOE‐7 probe, corresponding to the ancestral fusion of GGA‐9 with two microchromosomes, maintained that configuration without undergoing fission to produce IHA‐5. The BOE‐14 probe, typically fragmented in all cytogenomically characterized Passeriformes, corresponded to two distinct GGA segments: the R3 portion and a region of unknown homology. This is the first documentation of such chromosomal organization in passerines. The absence of these fissions suggests a possible autapomorphic chromosomal condition within Thamnophilidae. Additionally, a potential fission involving microchromosomes homeologous to probes BOE‐15 and BOE‐16 is suggested: while these probes mark three microchromosome pairs in GGA, they mark four pairs in *I. hauxwelli* —a feature not yet observed in other passerines. As most Thamnophilidae remain cytogenetically uncharacterized, systematic study of this family is essential to determine whether these rearrangements represent synapomorphies, autapomorphies, or convergent events.

## 1 Introduction

Passeriformes represent the most speciose order within the class Aves, comprising over 6000 recognized species—approximately 60% of all avian diversity (BirdLife International 2025). This order exhibits a near‐global distribution, occurring across all continents except Antarctica (Johansson et al. 2008). In the Amazon Basin alone, avian diversity exceeds 900 species, with Passeriformes accounting for more than 500 species (Billerman et al. 2024). The order demonstrates its highest species richness in tropical regions, with the Neotropics harboring a significant proportion of this diversity. Notably, the family Thamnophilidae—comprising approximately 200 insectivorous species occupying varied ecological niches—represents 15% of the Neotropical passerine richness (Ribas et al. 2021).

Phylogenetically, Thamnophilidae belongs to the suborder Tyranni (Suboscines), forming a well‐supported clade with Conopophagidae and Melanopareiidae (Ohlson et al. 2013). Within the family, three distinct subfamilies are recognized: Euchrepomidinae, Myrmotherinae, and Thamnophilinae, with the latter containing the highest species diversity (Bravo et al. 2012; Ohlson et al. 2013).

Among the genera within the subfamily Thamnophilinae, *Isleria* is the least speciose, comprising only two species (Bravo et al. 2012). This genus was erected following phylogenetic analyses of *Myrmotherula*, which revealed the non‐monophyletic nature of the latter. Molecular evidence supported the reclassification of two species— *Isleria guttata* and *Isleria hauxwelli* —into this newly defined genus (Bravo et al. 2012).

Despite the remarkable complexity of avian clades, cytogenetic data remain limited, with karyotypes described for only approximately 10% of bird species. Furthermore, comprehensive studies employing chromosome painting and in situ hybridization techniques—crucial for identifying chromosomal rearrangements that contribute to genome evolution—remain particularly scarce. These rearrangements are known to occur frequently in avian karyotypes. Among avian orders, Passeriformes represents the most extensively studied group in terms of classical cytogenetic analyses (Degrandi et al. 2020).

Current research on chromosome painting in birds has been facilitated by whole chromosome probe sets developed for several species: *Gallus gallus* (GGA, 2n = 78, Galliformes; Griffin et al. 1999), *Burhinus oedicnemus* (BOE, 2n = 42, Charadriiformes (Nie et al. 2009)), *Leucopternis albicollis* (LAL, 2n = 66; de Oliveira et al. 2010), *Gyps fulvus* (GFU, 2n = 66; Nie et al. 2015), *Zenaida auriculata* (ZAU, 2n = 76; Kretschmer, de Oliveira Furo, et al. 2018), and *Myiopsitta monachus* (2n = 48; Furo et al. 2020). Among these, only *G. gallus* has a fully sequenced genome, owing to its significant economic importance (Warren et al. 2010). Additionally, the *G. gallus* karyotype exhibits high conservation relative to the hypothesized ancestral avian karyotype, making it a pivotal reference for comparative genomic studies (Griffin et al. 2007; Ribas et al. 2021). Consequently, *G. gallus* probes have been extensively utilized in avian cytogenetic research. Regarding the BOE probes, this set of probes initially provided the study of microchromosomes in a more present way, since the GGA probes were limited to macrochromosomes and sex chromosomes, with the BOE chromosome set consisting of 42 chromosomes, many of them homeologous to GGA microchromosomes (Nie et al. 2009; Pinheiro et al. 2022; Ribas et al. 2021; Ribas et al. 2018). This karyotype diverges from what is known as the avian ancestral karyotype both in total number of chromosomes and in the amount of microchromosomes in the BOE karyotype (Griffin et al. 2007). The implementation of the use of this new set of total chromosome probes provided an increase in resolution and analysis capacity for chromosomal study in birds, especially regarding microchromosomes.

The family Thamnophilidae exhibits an average diploid chromosome number (2n) approximating 80. However, cytogenetic data remain limited, with only a small fraction of species characterized to date. Currently available karyotypic information includes: *Dysithamnus mentalis* (2n = 76), *Pyriglena leucoptera* (2n = 82), *Willisornis vidua* (2n = 80), *Thamnophilus doliatus* (2n = 82) (de Lucca 1977; Ledesma et al. 2002; Ribas et al. 2021).

Several studies have reported rearrangements involving both microchromosomes and macrochromosomes in Passeriformes, although such events are relatively uncommon in birds. For example, *Tolmomyias sulphurencens* (2n = 60) presents two rearrangements involving the homeologs GGA13/micro and GGA28/micro (Kretschmer et al. 2021), the authors highlight that possibly microchromosome probes not used in the research may be the ones that merged with the homeologs GGA13 and GGA28 and gave rise to TSU10 and TSU11 in the target species of the study. Another example can be seen in the hybridization of BOE and GGA probes in the karyotype of *Willisornis vidua* (2n = 80), where the authors demonstrated the fusion between the BOE5 probe, homeologous to GGA7 (Nie et al. 2009), with a microchromosome and thus forming the WVI7 pair of the target species of the study.

Therefore, the present study seeks to explore the karyotypic study of the genus *Isleria* through the following targets: (1) characterize the karyotype of a single male specimen *Isleria hauxwelli* (IHA; Thamnophilidae, Passeriformes) using chromosome painting techniques, and (2) identify potential chromosomal rearrangements underlying its karyotypic evolution. These findings will provide crucial insights into the phylogenetic positioning of *I. hauxwelli* and contribute to our understanding of karyotype evolution within the Thamnophilidae family.

## 2 Methodology

### 2.1 Ethics Statement

JCP holds a permanent field permit number 13248 from “Instituto Chico Mendes de Conservação da Biodiversidade”. The Cytogenetics Laboratory from UFPA has special permits 19/2003 for sample transport and 52/2003 for research use, granted by the Brazilian Ministry of Environment. The Ethics Committee (Comitê de Ética Animal da Universidade Federal do Pará) approved this research (Permit 68/2015).

### 2.2 Obtaining Samples and Chromosomes

#### 2.2.1 Sampling

A single male specimen of *Isleria hauxwelli* was collected during field surveys conducted at Gunma Ecological Park (1°12′05.0′′ S, 48°17′50.7′′ W) in Santa Bárbara do Pará municipality, Pará state, Brazil.

Specimen collection was performed using standardized mist netting techniques (CEMAVE 1994), with 12 × 2 m nets arranged linearly to form an interception barrier. Nets were monitored at 30‐min intervals to minimize stress on captured individuals. To enhance capture efficiency, we employed acoustic luring techniques using playback of conspecific vocalizations broadcast via portable audio equipment.

#### 2.2.2 Chromosome Preparation Protocol

Chromosome preparations were obtained following the technique of Ford and Hamerton (1956) (Centrifuge for 7 min, remove the supernatant and add approximately 6 mL of hypotonic solution (0.075 M KCl), resuspend and incubate in an oven at 30°C for 20–30 min; Add approximately 15 drops of chilled 1:4 Acetic‐alcohool). The specimen first received an intraperitoneal injection of 0.05% aqueous colchicine solution at a dosage of 0.01 mL per 10 g body weight (in vivo colchicine treatment). This mitotic arrest procedure was maintained for 40–60 min prior to euthanasia.

Euthanasia was performed via intraperitoneal administration of lidocaine (20 mg/mL) at overdose concentrations. Following confirmation of death, the femur was aseptically excised using surgical instruments and immediately placed in a sterile Petri dish containing 5 mL of 0.075 M potassium chloride (KCl) hypotonic solution. The epiphyses were sectioned and bone marrow was flushed using repeated washes with hypotonic solution.

The harvested marrow was mechanically disaggregated using a tissue homogenizer to obtain a single‐cell suspension. Cells were then incubated in hypotonic solution at 37°C for 20–30 min to achieve optimal chromosome spreading. Following hypotonic treatment, 1 mL of ice‐cold Carnoy's fixative (3:1 methanol: glacial acetic acid) was added for prefixation.

The cell suspension was centrifuged at 1000 rpm for 7 min, after which the supernatant was carefully decanted. The pellet was gently resuspended in 5 mL fresh fixative using a Pasteur pipette. This fixation cycle was repeated twice for complete cellular preservation. Final cell concentration was adjusted by adding an appropriate volume of fixative to achieve optimal metaphase spread density for chromosomal analysis.

### 2.3 Conventional Chromosomal Staining Protocol

For conventional cytogenetic analysis of mitotic and meiotic cells, fixed cellular material was deposited onto clean microscope slides and subjected to Giemsa banding. The staining procedure was performed as follows: Slides were immersed in a 5% Giemsa solution (Merck, Darmstadt, Germany) prepared in phosphate buffer (pH 6.8; 10.6794 g of Na_2_HPO_4_. 2H_2_O: 8.1654 g of KH_2_PO_4_ for 1 L of distilled water, pH 6.8), for 5–8 min at room temperature (25°C ± 2°C) to achieve optimal chromosome banding patterns. Following staining, slides were rinsed gently with distilled water to remove excess dye.

### 2.4 Chromosome Painting Protocol

For comparative genomic analyses, we employed whole‐chromosome painting probes derived from *Burhinus oedicnemus* (Charadriiformes). These probes were generated through flow‐sorted chromosome isolation from cell cultures (Nie et al. 2009), and it is useful for presenting many microchromosomes fused in blocks, which allows us to directly compare these microchromosomes between karyotypes.

### 2.5 Fluorescence In Situ Hybridization (FISH) Protocol

The chromosomal homology analysis was performed using fluorescence in situ hybridization (FISH) with both single and double hybridization approaches. Probes were differentially labeled with either biotin (detected using Cy3‐conjugated avidin) or digoxigenin (detected with fluorescein‐conjugated anti‐digoxigenin antibodies), enabling simultaneous visualization of multiple chromosomal regions. Following hybridization, chromosomes were counterstained with DAPI (4′,6‐diamidino‐2‐phenylindole) for general chromatin identification, following the standard protocol established by Yang et al. (1997).

### 2.6 Microscopy and Image Acquisition

The chromosomal preparations were examined under a Carl Zeiss Axio Imager. D2 research‐grade microscope, equipped with an Axiocam 503 monochrome digital camera for high‐resolution imaging. Image acquisition was performed using Zeiss Axiovision 3.1 software. Subsequently, the images were optimized for publication through careful editing and enhancement using Adobe Photoshop CS6 software, ensuring accurate representation of the chromosomal features while maintaining scientific integrity.

## 3 Results

### 3.1 Karyotypic Characterization of *Isleria hauxwelli*

Cytogenetic analysis revealed that *I. hauxwelli* possesses a diploid chromosome number of 2n = 84, with a fundamental number (FN) of 92. The karyotype consists of 42 pairs of chromosomes found in the species; 33 pairs correspond to microchromosomes, with only 11 pairs of autosomal macrochromosomes, in addition to the pair of sex chromosomes. From pair 1 to pair 5, we can see subtelocentric chromosomes. Pairs 6, 7, and 8 were acrocentric. The Z chromosome was acrocentric, and the rest of the karyotype (pairs 12–41) was composed of microchromosomes (Figure 1).

**FIGURE 1:**
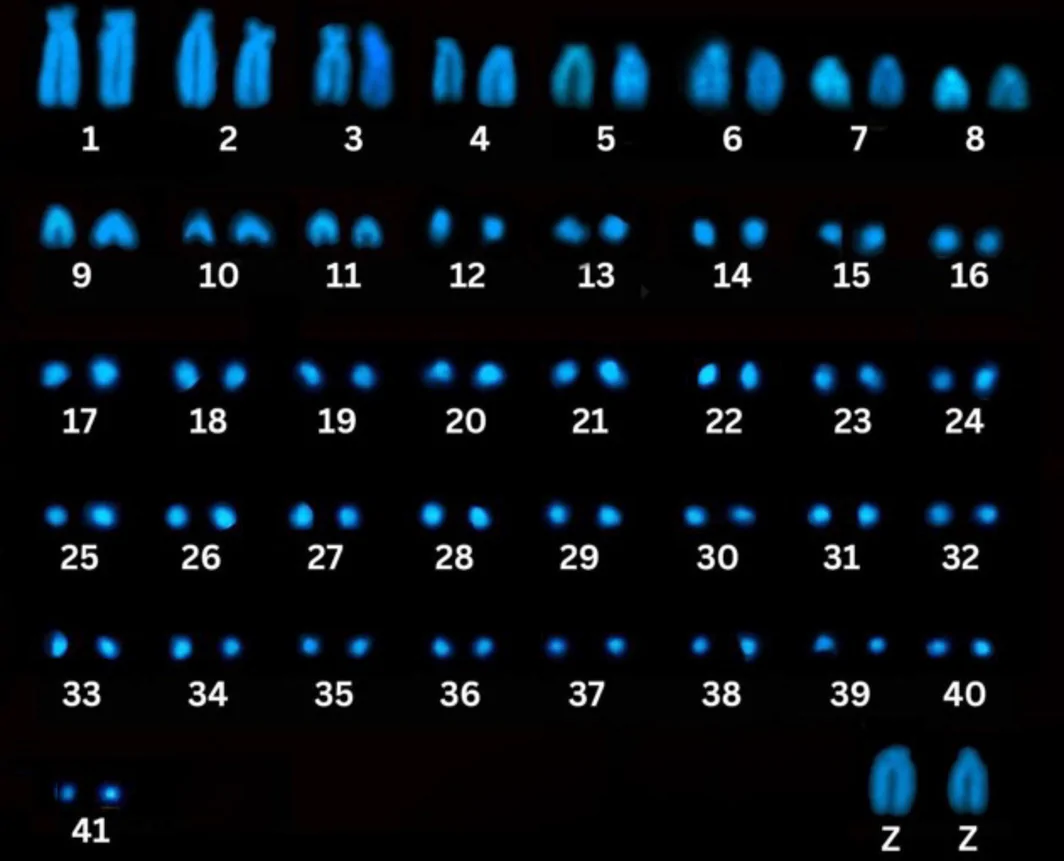
Conventional karyotype of male *Isleria hauxwelli* showing 2n = 84 chromosomes stained with DAPI (4′,6‐diamidino‐2‐phenylindole).

### 3.2 Comparative Genomic Analysis via Chromosome Painting

Fluorescence in situ hybridization (FISH) with *Burhinus oedicnemus* (BOE) whole‐chromosome probes revealed both conserved synteny and rearranged segments in *Isleria hauxwelli* (IHA) by indirect comparison with *Gallus* (GGA) karyotype (Nie et al. 2009). BOE‐1 and 2 probes, homeologs to GGA 1 and 2, respectively (Table 1), presented rearrangements between the species. BOE‐1 marked two different chromosome pairs (IHA‐1 and IHA‐6), as did BOE‐2 (IHA‐3 and IHA‐7); the chromosome probes BOE‐3 and 5, homeologs to GGA‐3 and 4q chromosomes, are conserved in *I. hauxwelli* . The BOE‐7 probe, homeolog to GGA‐5 chromosome, marked the IHA‐5 pair (Figures 2 and 5); BOE‐10 probe, homeolog to 2 GGA microchromosomes, marked IHA‐8 (Figures 2 and 5); and BOE‐4 probe, homeolog to GGA‐Z, marked IHA‐Z (Figures 2 and 4). The other probes marked microchromosomes (Figure 2). It was not possible to determine the results of BOE‐W, homeolog to GGA‐W, because the sample consisted of a male specimen.

**TABLE 1:** Chromosomal correspondence between *Burhinus oedicnemus*, *Gallus gallus*, and *Isler*ia hau*xwelli*.

Probe	Chromosome		
BOE	*Burhinus oedicnemus*	*Gallus gallus*	*Isleria hauxwelli*
1	1	1	1,6
2	2	2	3,7
3	3	3	2
5	4	4q	4
14	5	7, 8	—
12	6	5	2 mic
6	7	9, 2 mic	5
11	8	4p, 1 mic	—
17	9	6, 1 mic	3 mic
16	10	2 mic	1 mic
9	11	2 mic	2 mic
13	12	2 mic	2 mic
20	13	2 mic	2 mic
10	14	2 mic	8
8	15 + 16	3 mic	4 mic
7	17 + 18 + 19 + 20	1 mic	—
4	Z	Z	Z
15	W	Z, Zqter	—

*Note: Gallus gallus* and *Isleria hauxwelli* . The 5, 8, and 17 + 18 + 19 + 20 corresponding probes were not used in the study. *Source:* Nie et al. (2009); Author.

**FIGURE 2:**
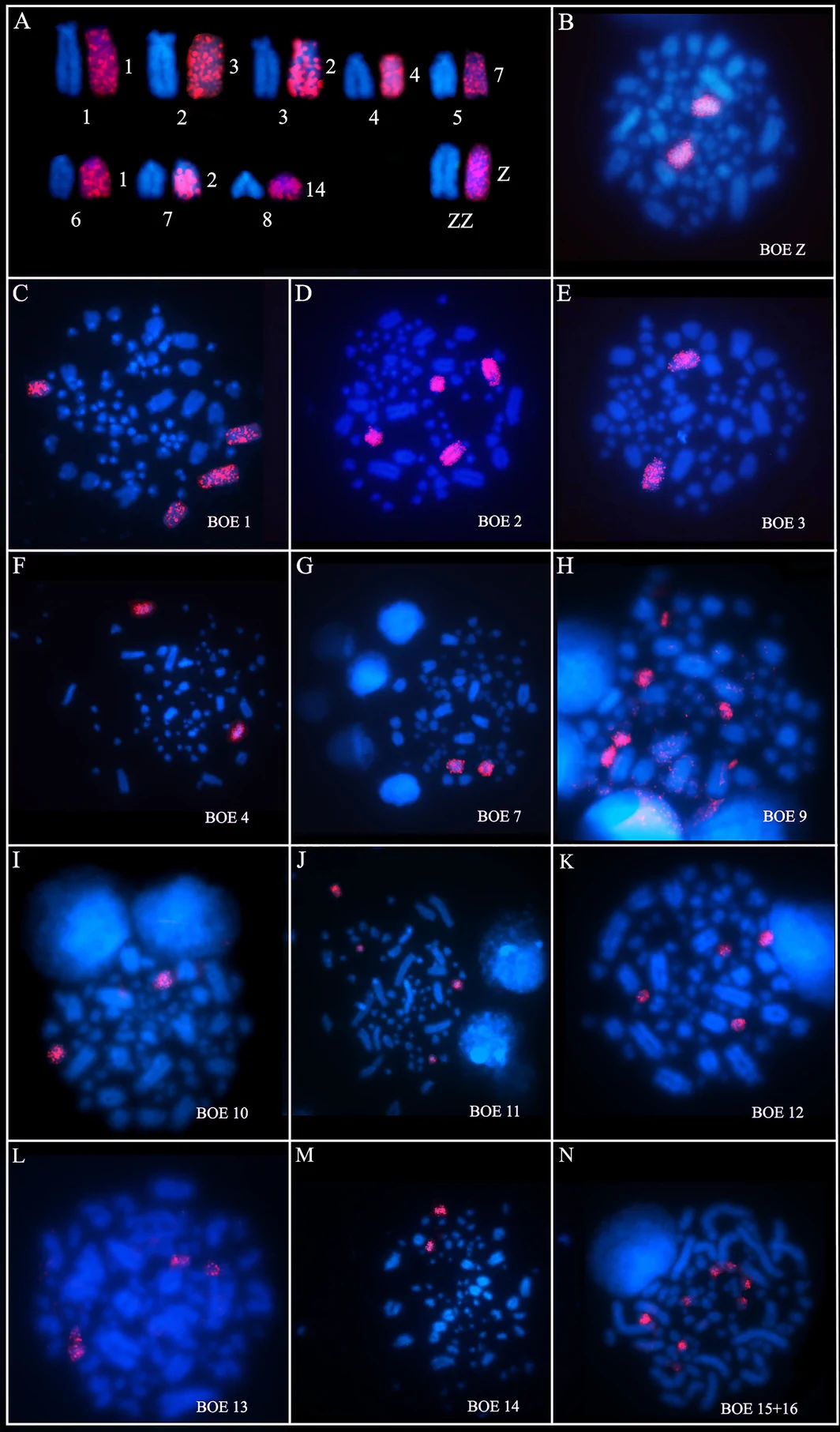
Comparative chromosome painting of *Isleria hauxwelli* (IHA) macrochromosomes and microchromosomes using *Burhinus oedicnemus* (BOE) whole‐chromosome probes. (A) The numbers located below the chromosomes refer to the IHA karyotype. The numbers located to the right refer to the BOE chromosomes. (B–N) The IHA metaphases with their corresponding BOE.

## 4 Discussion

We present the cytogenetic description of *Isleria hauxwelli* , revealing a diploid number of 2n = 84 (Figure 1), a karyotype common among Passeriformes and reminiscent of the putative ancestral avian karyotype (Griffin et al. 2007; Kretschmer, Ferguson‐Smith, and de Ferguson‐Smith 2018). However, *I. hauxwelli* exhibits distinct differences in chromosome morphology and structural rearrangements compared to other passerines.

Previously, *I. hauxwelli* was characterized by de Lucca (1977) with a diploid number of 80 chromosomes; however, in the present study, the new 2n was fixed at 84 chromosomes. This difference between the chromosome numbers presented by de Lucca (1977) and the present study may have been caused by limitations of the technique and technologies used by de Lucca (1977) at the time of its execution, in addition to the large number of microchromosomes presented in its karyotype, which may have increased the difficulty of the analysis.

While the fission of BOE‐1 and BOE‐2—resulting in their distribution across multiple chromosome pairs (IHA‐1/IHA‐6 and IHA‐3/IHA‐7, respectively) could be observed in some Passeriformes birds (dos Santos et al. 2015; Kretschmer et al. 2015; Ribas et al. 2021), this trait is not exclusive to the order. Similar rearrangements occur in other avian clades, including Strigiformes, Columbiformes, and Falconiformes (de Oliveira et al. 2008), suggesting either deep homology or convergent evolution of these genomic rearrangements.

### 4.1 Comparative Genomic Analysis of BOE‐1 and BOE‐2 Rearrangements

Our chromosome painting results revealed that the BOE‐1 probe (homeolog to GGA‐1 chromosome) hybridized to two distinct chromosome pairs in *Isleria hauxwelli* (Figures 2A,C and 3A), consistent with a fission event of the ancestral macrochromosome. This rearrangement pattern represents a widespread genomic signature across multiple Suboscines in the families Thamnophilidae (Ribas et al. 2021; Ribas et al. in press), Furnaridae (Kretschmer, de Lima, et al. 2018; Ribas et al. 2018), Conopophagidae (de Oliveira et al. 2020), Tyrannidae (Rodrigues et al. 2018) and Rhynchocyclidae (Kretschmer et al. 2021). The BOE‐2 probe reveals this fission in the families (excluding Rhynchocyclidae) (Kretschmer et al. 2021; Rodrigues et al. 2026), though it is absent in Oscines (Ribas et al. in press). In *Isleria hauxwelli* , the BOE‐2 fission results in two distinct new chromosome pairs, as illustrated in Figures 2A,D and 3B.

**FIGURE 3:**
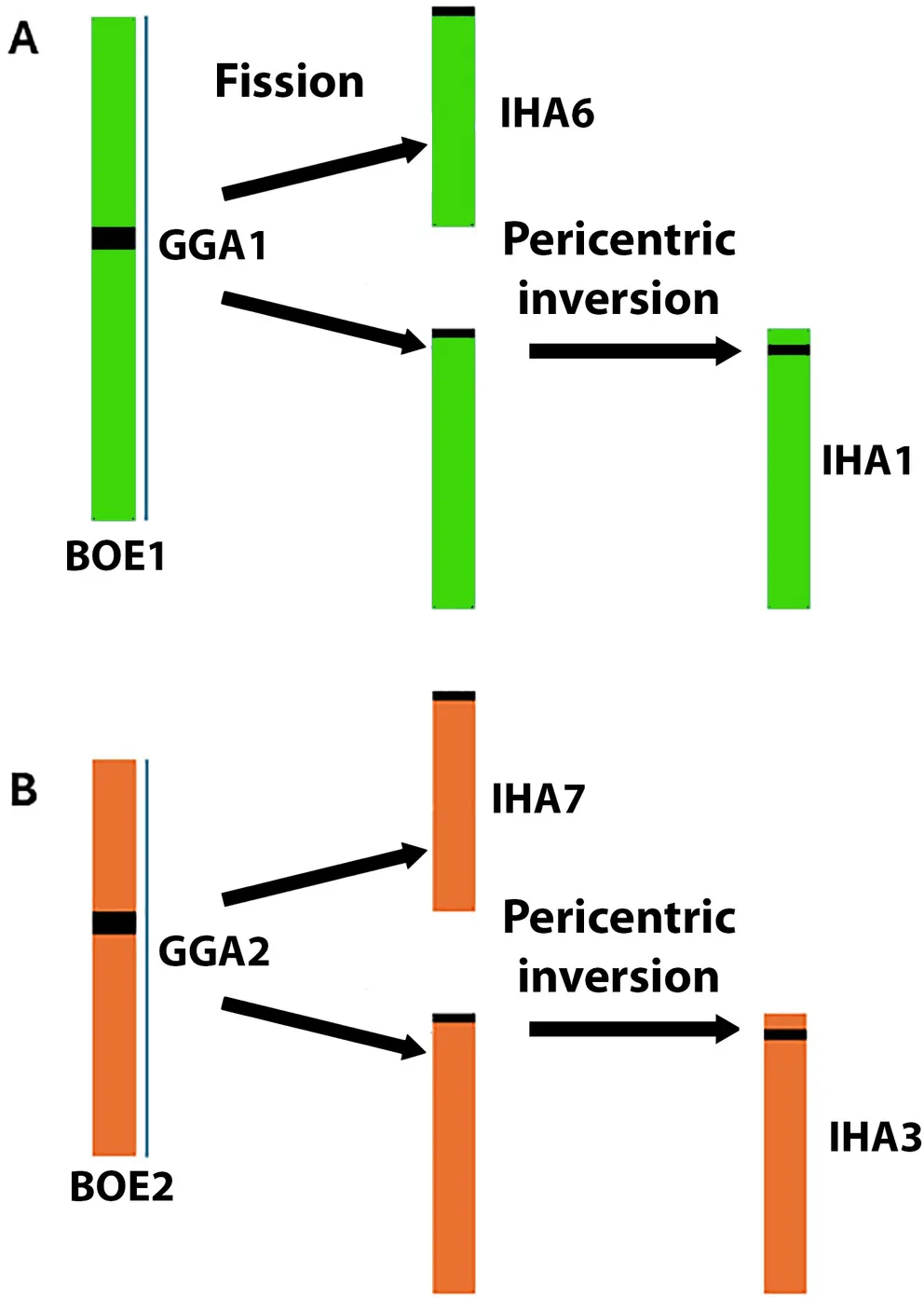
Schematic ideogram reconstructing macrochromosomal rearrangement events between *Burhinus oedicnemus* (BOE), *Gallus gallus* (GGA), and *Isleria hauxwelli* (IHA). (A) Chromosome fission in BOE‐1 (=GGA‐1) generating two chromosomes, followed by pericentric inversion in the formation of IHA‐1; (B) Chromosome fission in BOE‐2 (=GGA‐2) generating two chromosomes, followed by pericentric inversion in the formation of IHA‐3; Equivalence between BOE and GGA according to Nie et al. (2009).

### 4.2 Pericentric Inversion in BOE‐Z

In birds, sex chromosomes are generally the most stable elements within the karyotype, as they remain highly conserved across species, while autosomes frequently undergo various types of rearrangements—some more extensively than others. Passeriformes exhibit a pericentric inversion in the BOE‐Z homeologue (Nie et al. 2009), which has been documented across multiple species from various families, including *Turdus rufiventris* and *Turdus albicollis* (Turdidae), *Saltator aurantiirostris* and *Saltator similis* (Thraupiidae), *Willisornis vidua* (Thamnophilidae), *Glyphorynchus spirurus* (Furnariidae), and *Conopophaga lineata* (Conopophagidae), among others (de Oliveira et al. 2020; dos Santos et al. 2015; Kretschmer et al. 2014; Ribas et al. 2021; Ribas et al. 2018). The presence of this pericentric inversion led to the formation of the IHA‐Z (Figures 2A,B and 4), which is observed in the species examined in this study and shows consistency with other documented species. Another hypothesis for the change in the position of the centromere would be centromeric replacement, where the centromere would reorganize itself in another region of the chromosomal arm and give rise to the new centromere, without the need for a pericentric inversion. More cytogenetic studies are needed, with BAC‐FISH and G‐banding approaches to better understand this Z chromosome conformation in *Isleria hauxwelli* .

**FIGURE 4:**
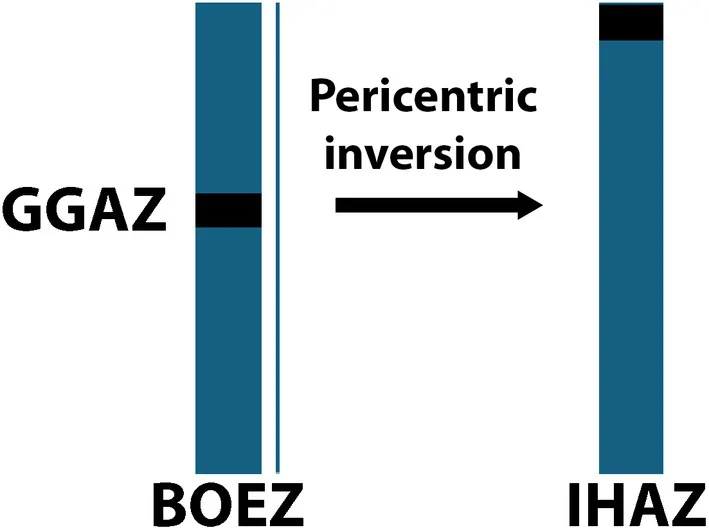
Sex chromosome evolution through pericentric inversion of BOE‐Z in the formation of IHA‐Z. Equivalence between BOE and GGA according to Nie et al. (2009).

### 4.3 BOE‐7 and 14

The BOE‐7 probe, corresponding to the ancestral fusion of GGA‐9 with two microchromosomes (Nie et al. 2009), may be an example of convergent evolution, since *Isleria hauxwelli* and *Burhinus oedicnemus* do not share a recent common ancestor, but rather present similar chromosomal characteristics that originated the IHA‐5 chromosome in *I. hauxwelli* and BOE‐7 in *Burhinus oedicnemus* (Figure 5A; Table 1), despite their homeologous relationship—an exceptional pattern among Passeriformes where this unfused state represents the typical chromosomal arrangement. The BOE‐14 probe (homeologs to two pairs of GGA microchromosomes), similar to BOE‐7, exhibits fragmentation across all citogenomically characterized Passeriformes (Figure 5B; Table 1), corresponding to two distinct GGA segments: the R3 portion and a region of undefined homology (Nie et al. 2009). Notably, this represents the first documentation of such chromosomal organization in passerines. Interestingly, an analogous conservation pattern of BOE‐7 has been reported in Charadriiformes species ( *Charadrius collaris* and *Vanellus chilensis* ) (Pinheiro et al. 2022), suggesting potential evolutionary parallels between these avian orders.

**FIGURE 5:**
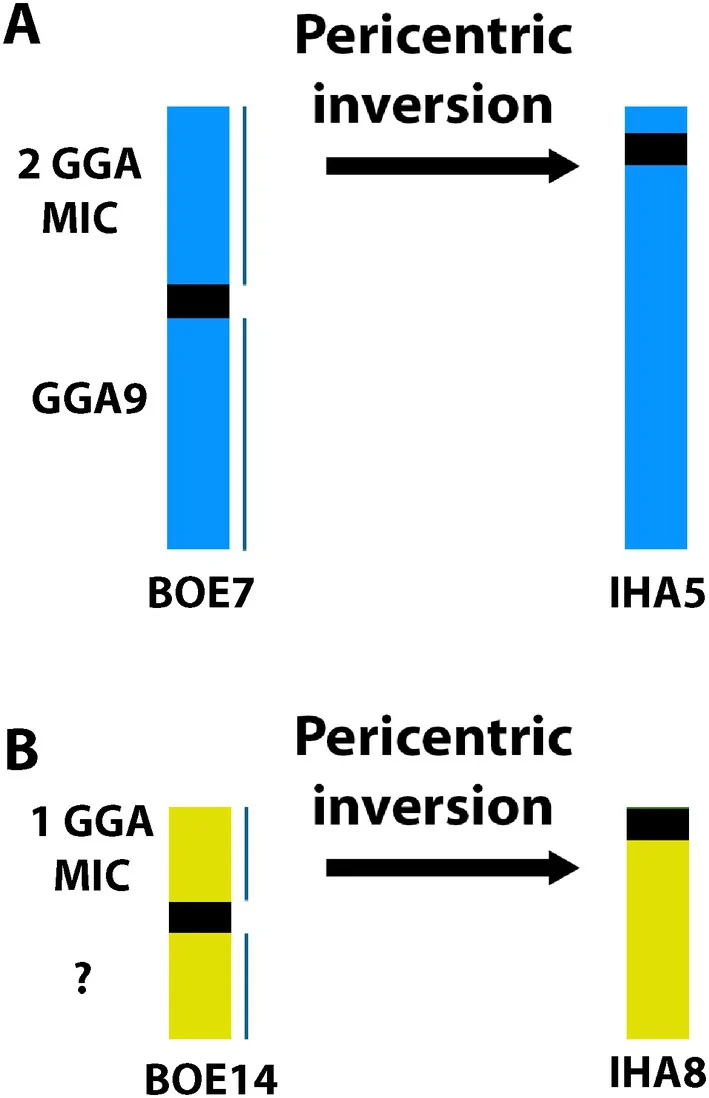
Pericentric inversion events in *Isleria hauxwelli* macrochromosome evolution. (A) Rearrangement of BOE‐7 (GGA‐9 homologue); (B) Pericentric inversion of BOE‐14 (=GGA mic) in the formation of IHA‐8. Equivalence between BOE and GGA according to Nie et al. (2009).

Comparative cytogenomics analyses demonstrate that multiple Passeriformes lineages (Turdidae, Thraupiidae, Thamnophilidae, Furnariidae, and Conopophagidae) exhibit conserved fission patterns in both BOE‐7 and BOE‐14 during karyotype evolution (de Oliveira et al. 2020; dos Santos et al. 2015; Kretschmer et al. 2014; Ribas et al. 2021; Ribas et al. 2018). The absence of these fissions in our study genus suggests a potential autapomorphic chromosomal condition within Thamnophilidae, though comprehensive sampling across the family is required to confirm this evolutionary scenario and exclude possible lineage‐specific reversals or convergent evolution. Figure 6 illustrates these rearrangements within a phylogenetic framework.

**FIGURE 6:**
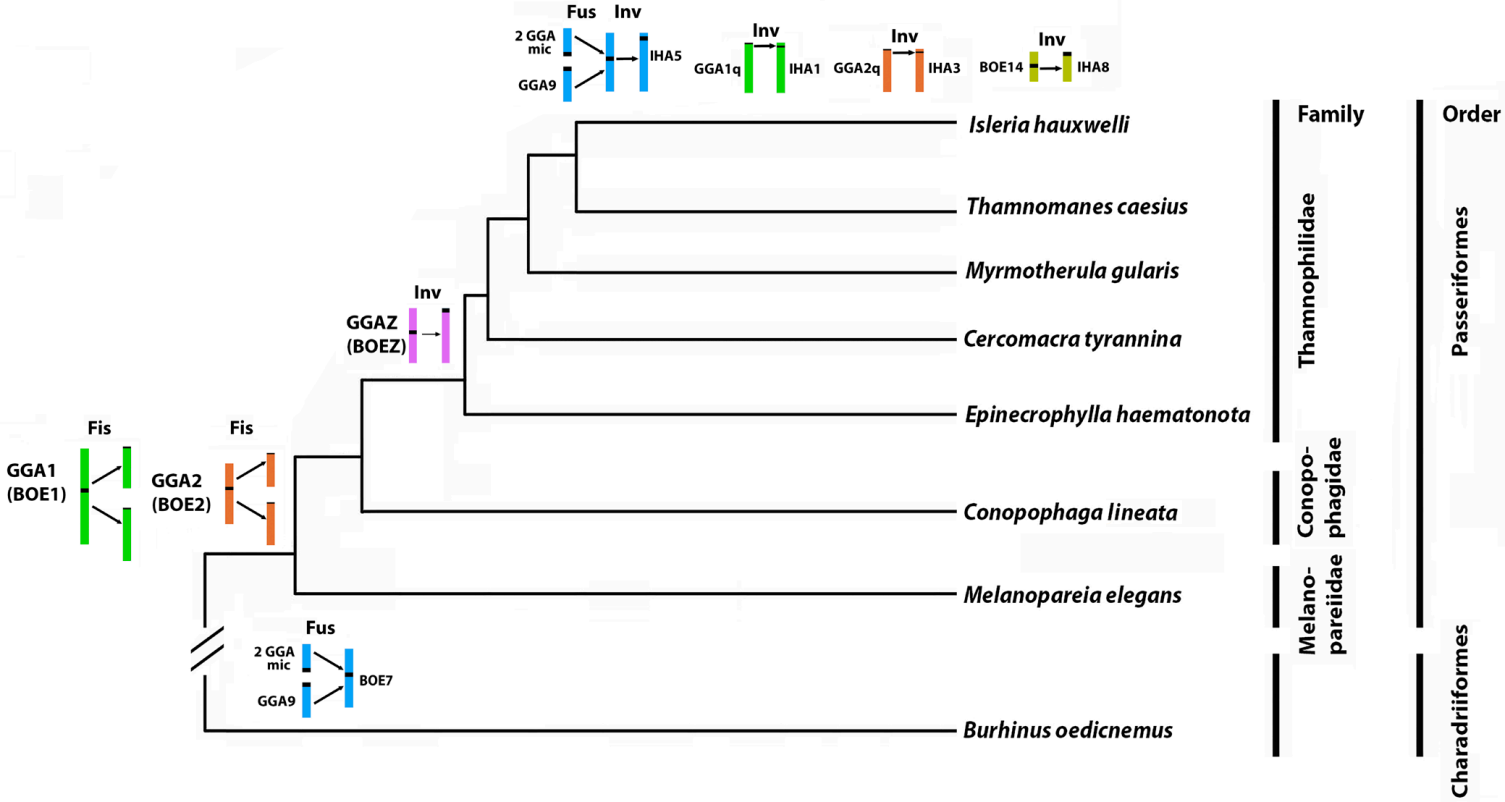
A simplified phylogeny of Passeriformes, based on Bravo et al. (2012) and Ohlson et al. (2013), with the representation of the rearrangements described in this article. Fus = fusion, Fis = fission, Inv = inversion; m = microchromosomes, q = long arm of the chromosome. The species where chromosome painting was made are *Conopophaga lineata* (de Oliveira et al. 2020), *Burhinus oedicnemus* (Nie et al. 2009), and *Isleria hauxwelli* (present work).

### 4.4 Possible Rearrangement Involving Break Points on Microchromosomes

Fusions between microchromosomes and macrochromosomes are relatively uncommon in birds, including Passeriformes, although such rearrangements have been documented in both birds generally and passerines specifically (de Oliveira et al. 2020; Kretschmer et al. 2014, 2021). In contrast, reports of microchromosome fissions are far rarer. To date, this type of chromosomal break has been described for only one species, *Veniliornis spilogaster* (2n = 88, Piciformes) (Alves Barcellos et al. 2024). In that study, BAC probes from *Gallus gallus* and *Taeniopygia guttata* were used in fluorescence in situ hybridization, revealing a fusion between GGA12, a microchromosome, and the second‐largest chromosome of *V. spilogaster* , followed by a rearrangement that split the microchromosomal region. This finding is notable because bird microchromosomes are generally considered highly resistant to breakage due to their high gene density and low frequency of breakpoints (Alves Barcellos et al. 2024). Likewise, (O’Connor et al. 2019), using BAC probes from *G. gallus* and *T. guttata* in 24 bird species from different orders, showed that microchromosomes are highly conserved. They also found that species with high diploid numbers, such as *Scolopax rusticola* (2n = 96, Charadriiformes), typically derive these counts from macrochromosome fissions rather than breaks involving microchromosomes, reinforcing the idea that microchromosomes are resistant to fragmentation. However, in the present study, the BOE15 and BOE16 probes hybridized to four pairs of microchromosomes in *Isleria hauxwelli* , whereas in *G. gallus* these probes label only three pairs (Nie et al. 2009). This pattern may indicate a chromosomal break involving microchromosomes and, if confirmed, would represent the first such finding in Passeriformes. Because this type of rearrangement appears to be extremely rare in birds, further studies are needed to confirm and better understand this result.

## 5 Conclusion

Chromosomal rearrangements in Passeriformes, particularly within Thamnophilidae, have been previously documented, with species like *Willisornis vidua* exhibiting fissions (e.g., in chromosome 1) like those observed in *Isleria hauxwelli* —not being a specific trait of the Thamnophilidae family. However, our study reveals novel, previously unreported rearrangements involving chromosomes IHA‐5 (homeologous to pairs of GGA‐9 and one of microchromosome) and IHA‐8 (homeologous to two pairs of GGA microchromosomes), which appear unique among all examined passerine species to date. Our findings represent the first report of such chromosomal rearrangements within Passeriformes, underscoring the need for expanded comparative studies across the order. In addition, the presence of possible chromosomal breaks involving microchromosomes (BOE15 + 16 Probe, homeologs to three pairs of GGA microchromosomes) may be a unique finding among Passeriformes and needs special attention in the chromosomal study of birds. To assess the evolutionary significance of these traits, we propose: (1) chromosome painting with BOE probes in additional *Isleria* species to test for genus‐wide conservation; and (2) karyotypic analysis of the sister genus *Myrmothrula* to evaluate whether this characteristic reflects phylogenetic relationships. Given that most Thamnophilidae species remain cytogenetically uncharacterized, systematic investigation of this family is critical to determine whether these rearrangements constitute synapomorphies, autapomorphies, or convergent evolutionary events.

## Author Contributions

**Paulo Victor De Moraes Ferreira:** conceptualization, data curation, formal analysis, writing – original draft, visualization, methodology. **Talita Fernanda Augusto Ribas:** conceptualization, data curation, formal analysis, visualization, writing – review and editing, methodology. **Ane Nobre Monteiro:** methodology, formal analysis, investigation, writing – review and editing. **Lucas Guilherme de Silva Tembra:** formal analysis, methodology, visualization, writing – review and editing. **Milla de Andrade Machado:** methodology, investigation, formal analysis, writing – review and editing. **Patricia C. M. O'Brien:** methodology, visualization, writing – review and editing. **Malcolm Andrew Ferguson‐Smith:** resources, formal analysis, writing – review and editing, funding acquisition, methodology, validation. **Fengtang Yang:** methodology, resources, visualization, writing – review and editing. **Cleusa Yoshiko Nagamachi:** investigation, funding acquisition, methodology, resources, writing – review and editing. **Julio Cesar Pieczarka:** conceptualization, funding acquisition, investigation, methodology, project administration, resources, writing – review and editing, supervision.

## Funding

This work was supported by Coordenação de Aperfeiçoamento de Pessoal de Nível Superior, 047/2012. Banco Nacional de Desenvolvimento Econômico e Social, 2.318.697.0001. Conselho Nacional de Desenvolvimento Científico e Tecnológico, 307170/2021‐7, 307154/2021‐1.

## Conflicts of Interest

The authors declare no conflicts of interest.

## Data Availability

The data that supports the findings of this study are available at the article.
